# *EGFR* Mutation and 11q13 Amplification Are Potential Predictive Biomarkers for Immunotherapy in Head and Neck Squamous Cell Carcinoma

**DOI:** 10.3389/fimmu.2022.813732

**Published:** 2022-03-16

**Authors:** Shengjin Dou, Lin Zhang, Chong Wang, Yanli Yao, Wen Jiang, Lulu Ye, Jiang Li, Sicheng Wu, Debin Sun, Xiaoli Gong, Rongrong Li, Guopei Zhu

**Affiliations:** ^1^Department of Oral and Maxillofacial-Head Neck Oncology, Shanghai Ninth People’s Hospital, Shanghai Jiao Tong University School of Medicine, College of Stomatology, Shanghai Jiao Tong University, National Center for Stomatology, National Clinical Research Center for Oral Diseases, Shanghai Key Laboratory of Stomatology, Shanghai, China; ^2^Department of Oral Pathology, Shanghai Ninth People’s Hospital, College of Stomatology, Shanghai Jiao Tong University School of Medicine, Shanghai, China; ^3^Biostatistics Office of Clinical Research Center, Shanghai Ninth People’s Hospital, Shanghai Jiao Tong University School of Medicine, Shanghai, China; ^4^Department of Medicine, Genecast Biotechnology Co., Ltd, Wuxi, China

**Keywords:** head and neck squamous cell carcinoma (HNSCC), immune checkpoint inhibitor (ICI), predictive biomarkers, immunotherapy, 11q13 amplification, EGFR mutation

## Abstract

**Background:**

Head and neck squamous cell carcinoma (HNSCC) is one of the most common malignant cancers. The treatment of HNSCC remains challenging despite recent progress in targeted therapies and immunotherapy. Research on predictive biomarkers in clinical settings is urgently needed.

**Methods:**

Next-generation sequencing analysis was performed on tumor samples from 121 patients with recurrent or metastatic HNSCC underwent sequencing analysis. Clinicopathological information was collected, and the clinical outcomes were assessed. Progression-free survival (PFS) was estimated using the Kaplan-Meier method and cox regression model was used to conduct multivariate analysis. Fisher’s exact tests were used to calculate clinical benefit. A p value of less than 0.05 was designated as significant (p < 0.05).

**Results:**

Chromosome 11q13 amplification (*CCND1*, *FGF3*, *FGF4*, and *FGF19*) and *EGFR* mutations were significantly associated with decreased PFS and no clinical benefits after treatment with a programmed death 1 (PD-1) inhibitor. The same results were found in the combined positive score (CPS) ≥ 1 subgroup. In patients who were treated with an EGFR antibody instead of a PD-1 inhibitor, a significant difference in PFS and clinical benefits was only observed between patients with CPS ≥ 1 and CPS < 1.

**Conclusion:**

Chromosome 11q13 amplification and *EGFR* mutations were negatively correlated with anti-PD-1 therapy. These markers may serve as potential predictive biomarkers to identify patients for whom immunotherapy may be unsuitable.

## Introduction

Head and neck squamous cell carcinoma (HNSCC) is the sixth most common malignant cancer worldwide (1), accounting for 450,000 deaths per year (2). Treatment for HNSCC typically includes surgery, radiotherapy, chemotherapy, or combined modalities, and more recently, targeted therapy and immunotherapy. Compared to traditional therapies, both targeted therapy and immune checkpoint inhibitor (ICI) therapy have shown significantly improved clinical benefits (CBs) in the treatment of recurrent and metastatic disease (3–6).

ICIs that target the programmed death 1 (PD-1) and PD ligand 1 (PD-L1) axis, such as nivolumab and pembrolizumab, have been widely used and have proven to be effective in solid tumors. In 2016, based on the clinical trials CheckMate141 and KEYNOTE-012, these drugs were approved for use as second-line treatments in patients with recurrent or metastatic HNSCC that was refractory to platinum-based therapy (7, 8). In 2019, pembrolizumab was approved by the US Food and Drug Administration as a first-line treatment for patients with metastatic or unresectable recurrent HNSCC with a combined positive score (CPS) ≥1, based on the clinical trial KEYNOTE-048 (3).

However, successful ICI therapy generally requires the identification of clinically effective biomarkers with which to screen potential patients for treatment sensitivity. Currently, widely accepted pancancer biomarkers such as microsatellite instability, tumor mutation burden, and PD-L1 expression are used to identify patients who could potentially benefit from ICI treatment. Nonetheless, immunotherapy is applicable in a very limited proportion of patients (approximately 18%) (7, 9), leaving the vast majority vulnerable to trial and error methods for ICI treatment. More predictive biomarkers are needed to improve the quality of care for the unresponsive majority. Furthermore, there is a need to create protocols for the stratification of patients based on predictive biomarkers in order to apply personalized treatment at a clinical level.

Recent genomic and transcriptomic investigations have substantially improved our knowledge of the molecular mechanisms underpinning HNSCC (10, 11). Genetic aberrations and the abnormal expression of certain genes, such as *EGFR* (12, 13) and 11q13 amplification (CCND1, FGF3, FGF4, and FGF19) (14, 15) are closely associated with prognosis and may be useful prognostic biomarkers. In 2017, Singavi et al. identified *EGFR* and 11q13 amplification to be potential predictive biomarkers, as these genetic alterations were associated with hyper-progression in response to ICI in five patients with lung, esophageal, and renal cancers (16). Nevertheless, research on their roles as predictive biomarkers remains limited, especially in HNSCC. To meet the urgent need for more predictive biomarkers that can guide clinical decision-making, the aim of our retrospective study was therefore to explore the predictive roles of these genetic abnormalities in identifying patients who are unsuitable for immunotherapies in the real world.

## Materials and Methods

### Patients

One hundred and twenty-one patients were enrolled in the study from January 9, 2019, to November 10, 2020. The main inclusion criteria were as follows: (a) Pathology confirmed recurrent or metastatic HNSCC treated with anti-PD-1 antibody or EGFR antibody; (b) an age of 18 to 80 years; (c) no serious comorbidities (e.g., had suffered from other malignant tumors); (d) complete and usable follow-up data. Two cohorts of patients were enrolled: a PD-1 cohort that included 98 patients who were treated with anti-PD-1 antibody (PD-1 group), and a non-PD-1 cohort (NPD-1 group) that included 32 patients who were treated with non-PD-1 inhibitor (EGFR antibody). This study was approved by the ethics committee of Shanghai Ninth People’s Hospital (No. SH9H-2020-T257-1). Clinicopathological data were collected from patients during treatment and follow-up visits.

### Assessment of Clinical Benefit

In Assessment of clinical benefit, we referred to Yu et al. (17). Clinical efficacy was evaluated per RECIST 1.1 every 8 weeks. CB was defined as a patient exhibiting a complete response (CR) or partial response (PR) according to RECIST 1.1 (i.e., tumor shrinkage > 30% from baseline) or stable disease (SD) if they had any objective reduction in tumor burden lasting at least 6 months. No clinical benefit (NCB) were defined as those experiencing progressive disease according to RECIST 1.1 or SD lasting <6 months and were discontinued from immunotherapy within 3 months (18, 19).

### Targeted Sequencing of Clinical Samples

Tumor genomic DNA was isolated from formalin-fixed paraffin-embedded (FFPE) tissue sections using the QIAgen DNA FFPE tissue kit (Germantown, MD, USA), and was used for targeted sequencing with a cancer-related-gene panel (Genecast Biotech., Wuxi, China). Using Qiagen DNA blood mini kit, genomic DNA was extracted from peripheral blood collected from participants, and was used as a matched control. The library was constructed with 300 ng of genomic DNA from each participant. Fragment libraries were prepared from samples sheared by sonication, and target regions were enriched using customized IDT library prep kits (Integrated DNA Technologies, Coralville, IA, USA). The captured DNA was amplified, and the paired-end library was sequenced using the NovaSeq 6000 platform (Illumina, San Diego, CA, USA). Bioinformatics analysis was performed using an in-house program (Genecast Biotech.).

### Statistical Analysis

Statistical analyses were performed using MedCalc (version 19.0.4) (MedCalc Software Ltd, Ostend, Belgium). Progression-free survival (PFS) was estimated using the Kaplan−Meier method, and between-group differences in PFS were tested using the log-rank test. Cox regression model was used for multivariate analysis of PFS. Fisher exact tests were used to analyze the association between genetic aberrations with CBs. Odds ratios (ORs) and their associated 95% confidence intervals (CIs), and multivariate analysis of CB/NCB were estimated using the logistic regression model. A P value of less than 0.05 was considered statistically significant (p < 0.05).

## Results

### Patient Characteristics

Of the 121 patients enrolled in the study, 92 were male and 29 were female. The median age of the patients was 57 years. Ninety-one patients had oral squamous cell carcinoma, 23 had oropharyngeal carcinoma, and 7 had other cancer types. Assessment of PD-L1 status revealed that 84 patients had a CPS ≥ 1, 30 patients had a CPS < 1, and 7 patients had an unknown CPS status. The clinicopathological data are summarized in **Table 1**. The detailed clinicopathological data for each patient is shown in **Supplemental Table S1**.

**Table 1:** Summary of clinical information.

	Number	%
Sex		
Male	92	76.0%
Female	29	24.0%
Age (years)		
Median	57 (27–79)	
Cancer type		
Oral squamous cell carcinoma	91	75.2%
Oropharyngeal carcinoma	23	19.0%
P16 positive	5	4.1%
P16 negative	18	14.9%
Other cancer type	7	5.8%
PD-L1		
CPS < 1	30	25.0%
CPS ≥ 1	84	69.4%
Unknown	7	6.0%
Number of prior lines of therapy*		
0	70	NA
≥1	60	NA

*9 patients were treated with both PD-1 antibody and EGFR antibody at different lines. NA, not applicable.

### Overall Analysis of Patients Treated With PD-1 Antibody

**Figure 1:**
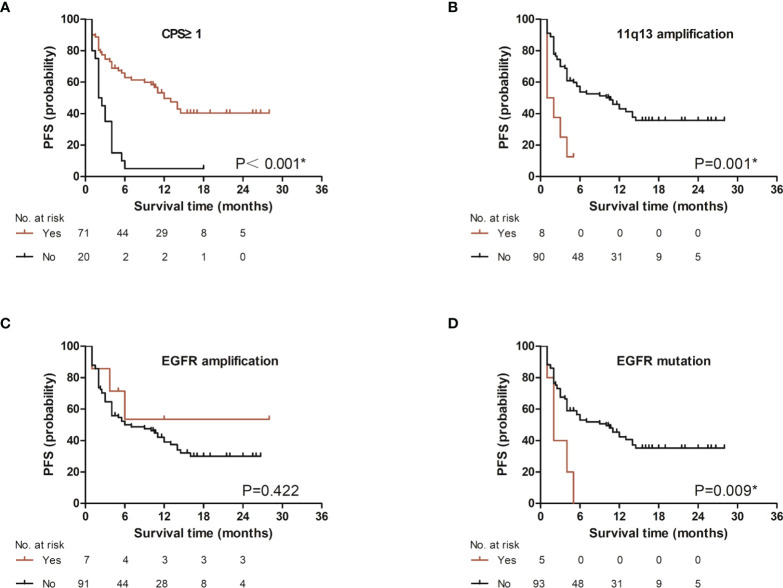
Association of several factors with progression-free survival in patients treated with PD-1 inhibitor. Association of **(A)** CPS level. (CPS, combined positive score), **(B)** 11q13 amplification status. (11q13: CCND1_FGF3_FGF4_FGF19 or any one of them), **(C)** *EGFR* amplification status, or **(D)** *EGFR* mutation status, with progression-free survival. *p<0.05.

**Figure 2:**
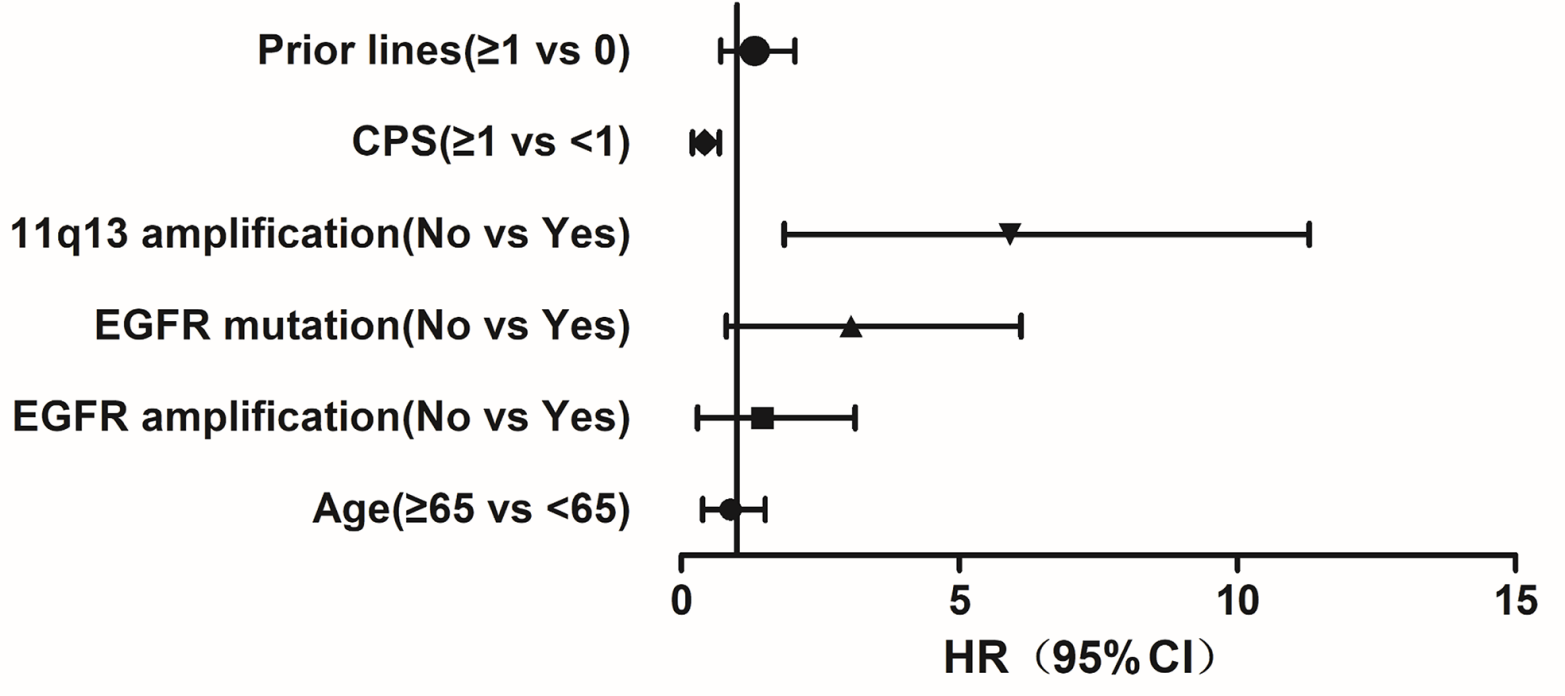
Multivariate analyses of treatment lines, CPS level, 11q13 amplification, *EGFR* mutation, *EGFR* amplification, and age with progression-free survival. CPS and 11q13 amplification are independent factor affecting progression-free survival.

We first analyzed the PFS based on CPS level (CPS ≥ 1 and CPS < 1), 11q13 status, *EGFR* amplification status and *EGFR* mutation status, we found that PFS was significantly different between the groups with different CPS (p < 0.001, **Figure 1A**). A significant difference in PFS was observed between the groups with different 11q13 amplification status (p = 0.001, **Figure 1B**). The PFS was not significantly different between the groups with different *EGFR* amplification status (p = 0.422, **Figure 1C**). A significant difference was observed between the groups with different *EGFR* mutation status (p = 0.009, **Figure 1D**). Multivariate analyses of treatment lines, CPS level, 11q13 amplification, *EGFR* mutation, *EGFR* amplification, and age showed that CPS and 11q13 amplification are independent factors affecting PFS with HR = 0.37 and 4.58, respectively (**Figure 2**).

We next evaluated whether CB was associated with CPS, 11q13 amplification, *EGFR* amplification, or *EGFR* mutations in patients who received anti-PD-1 treatment. Of the 71 patients with CPS ≥ 1, 46 (64.8%) benefited from anti-PD-1 treatment, while only three of the 20 patients with CPS < 1 obtained CB (p = 0.001; OR = 10.43; 95% CI: 2.78 - 39.05). Of the 90 patients without an 11q13 amplification, 51 (56.7%) obtained CB, while one of the 8 patients with an 11q13 amplification exhibited CB (p = 0.024; OR = 9.15; 95% CI: 1.08 - 77.52). Of the 93 patients with no *EGFR* mutations, 52 (55.9%) obtained CB, whereas none of the 5 patients with *EGFR* mutations obtained CB (p = 0.020; OR = 13.92; 95% CI: 0.75 – 258.94). Details are shown in **Table 2**. Multivariate analysis including treatment lines, CPS level, 11q13 amplification, *EGFR* mutation, *EGFR* amplification, and age showed only CPS level significantly associated with CB (p = 0.001).

**Table 2:** Logistic analysis of factors that affect clinical benefit.

Characteristic	Clinical benefit (n, %)		p value (Fisher exact test)	Odds ratio (95% CI)
	CB	NCB		
CPS				
≥1	46 (64.8)	25 (35.2)	<0.001	10.43 (2.78, 39.05)
<1	3 (15.0)	17 (85.0)		
11q13 amplification				
No	51 (56.7)	39 (43.3)	0.024	9.15 (1.08, 77.52)
Yes	1 (12.5)	7 (87.5)		
*EGFR* amplification				
No	48 (52.7)	43 (47.3)	1	0.84 (0.18, 3.95)
Yes	4 (57.1)	3 (42.9)		
*EGFR* mutation				
No	52 (55.9)	41 (44.1)	0.020	13.92 (0.75, 258.94)
Yes	0 (0)	5 (100)		

CB, clinical benefit; NCB, non-clinical benefit; CPS, combined positive score; 11q13: CCND1_FGF3_FGF4_FGF19 or any one of them.

### Analysis of Predictive Biomarkers in Patients From the CPS ≥ 1 Subgroup Treated With PD-1 Inhibitor

Focusing further on patients with CPS ≥1 who received anti-PD-1 treatment, analysis of PFS between groups with or without 11q13 amplification, *EGFR* amplification, *EGFR* mutations revealed that PFS was significantly different between the groups with or without 11q13 amplification (p < 0.001) and *EGFR* mutation (p = 0.022) (**Figure S1**). PFS was not significantly different between the groups with or without *EGFR* amplification (p = 0.990). Multivariate analyses of treatment lines, 11q13 amplification, *EGFR* mutation, *EGFR* amplification, and age showed that 11q13 amplification and *EGFR* mutation are independent factors affecting PFS with HR = 8.62 and 4.78, respectively (**Figure S2**).

We next evaluated whether 11q13 amplification, *EGFR* amplification, or *EGFR* mutation with CB. Our analysis indicated that 46 of 66 patients lacking an 11q13 amplification exhibited CB (69.7%), while none of the five patients with an 11q13 amplification demonstrated CB (p = 0.004; OR = 24.95; 95% CI: 1.32 - 472.62). Of the 65 patients with no *EGFR* amplification, 42 were evaluated as CB (64.6%), while four of the six patients with *EGFR* amplification exhibited CB (66.7%) (p=1.000; OR = 0.91; 95% CI: 0.16 - 5.37). The analysis of patients with no *EGFR* mutations showed that 42 of 69 exhibited CB (64.6%), whereas neither of the 2 patients with *EGFR* mutations demonstrated CB (0.00%) (p = 0.121; OR = 9.89; 95% CI: 0.46 - 214.55). *EGFR* mutations were not markedly associated with NCB in patients with CPS ≥1. Detailed information is shown in **Table 3**. Multivariate analyses show no factors are significantly associated with CB.

**Table 3:** Subgroup analysis of patients with CPS ≥ 1.

Characteristic	Clinical benefit (n, row%)		p value (Fisher exact test)	Odds ratio (95% CI)
	CB	NCB		
11q13 amplification				
No	46 (69.7)	20 (30.3)	0.004	24.95 (1.32, 472.62)
Yes	0 (0)	5 (100)		
*EGFR* amplification				
No	42 (64.6)	23 (35.4)	1.000	0.91 (0.16, 5.37)
Yes	4 (66.7)	2 (33.3)		
*EGFR* mutation				
No	46 (66.7)	23 (33.3)	0.121	9.89 (0.46, 214.55)
Yes	0 (0)	2 (100)		

CB, clinical benefit; NCB, non-clinical benefit; CPS, combined positive score; 11q13: CCND1_FGF3_FGF4_FGF19 or any one of them.

### Analysis of Predictive Biomarkers in Patients Treated With EGFR Antibody

To assess whether these predictive biomarkers were associated with PFS in patients treated with non-PD-1 inhibitor (EGFR antibody), we found only CPS level were associate with PFS in both univariate analysis (**Figure S3**, p = 0.030) and multivariate analysis (HR = 0.29). To assess whether these predictive biomarkers were associated with CB in non-PD-1 inhibitor treatment (EGFR antibody), we analyzed the non-PD-1 inhibitor group. In the subgroup analyses based on 11q13 amplification, *EGFR* amplification, and *EGFR* mutation status, no significant differences were observed between patients with and without mutations. However, significant differences were observed between patients with CPS ≥1 compared to those with CPS <1, indicating that CPS could predict CB even in patients who did not receive a PD-1 inhibitor. Detailed information is shown in **Supplemental Table S2**. Multivariate analyses show no factors are significantly associated with CB.

## Discussion

HNSCC is notorious for its high mortality rate, and frequent recurrence, and metastasis. While ICIs have broadened the clinical options for treating HNSCC, the successful application of these inhibitors requires the identification of predictive biomarkers that can help select for patients who are likely to exhibit CB. Here, we report the results of a retrospective, real-world study to determine the association between genetic aberrations and clinical outcomes.

The amplification of *EGFR* is not uncommon in HNSCC patients. *EGFR* has been identified as a predictive biomarker for chemotherapy or radiation/chemotherapy benefits and survival in oropharyngeal cancer and for targeted therapy benefit in HNSCC (20, 21). Prior to this study, little was known about the potential for *EGFR* amplification to predict immunotherapy benefit in HNSCC. We found that *EGFR* mutations, not *EGFR* amplification, adversely affected clinical outcomes when patients were treated with a PD-1 inhibitor ta. *EGFR* mutations were originally reported to affect the clinical outcomes of ICI treatment in TKI naïve, PD-L1-positive, and *EGFR*-mutant patients with advanced non-small-cell lung cancer (22). However, recent studies have indicated that the clinical outcomes of non-small-cell lung cancer patients with *EGFR* mutations are promising and that patients with different *EGFR* mutations may experience different outcomes of ICI therapy despite their generally low response to ICIs (23, 24). We observed that patients with *EGFR* mutations had a significantly decreased PFS and NCB to PD-1 inhibitors, which is in keeping with results from previous reports. Interestingly, when we further analyzed patients with CPS ≥ 1, we observed the same outcome in PFS, but not in CB., which may be due to the rare mutation rate in HNSCC. However, this observation requires further study because *EGFR* mutations are uncommon and the sample size in our study was small. *EGFR* overexpression was previously reported to be associated with poor PFS in HNSCC (13). In our study, however, *EGFR* amplification did not significantly affect clinical outcomes under any circumstances. This result is also different from that of a previous study which reported that 1 out of 26 patients with *EGFR* amplification who received ICI therapy experienced hyper-progression in different types of cancer (16). Further studies with large sample size are needed to validate the role of *EGFR* amplification in HNSCC.

The amplification of 11q13 has been observed in over 30% of patients with HNSCC and its prognostic value has also been reported (15, 25, 26). Their study indicated that higher expression of 11q13 amplification was indicative of worse prognosis. Chromosome 11q13 amplification was found to correlate significantly with carcinogenesis and the attenuation of effector immune cells in the tumor microenvironment (14). *CCND1* has been associated with resistance to PD-1-targeted therapy in a Chinese population with non-cutaneous melanoma due to its effects on innate immunity (17). In a retrospective study carried out at four French institutions, patients with recurrent and/or metastatic HNSCC experienced accelerated tumor growth and had shorter PFS after anti-PD-1 and anti-PD-L1 treatment, with three of the patients harboring a *CCND1* amplification (27). The role of *CCND1* amplification as a predictive biomarker for the efficacy of ICI therapy was also suggested in an in silico analysis performed by Chen et al. (14). In their study, the authors analyzed three large cohorts of solid tumors from The Cancer Genome Atlas (TCGA) database, the Memorial Sloan Kettering Cancer Center (MSKCC) archive, and a local database, and they concluded that *CCND1* amplification correlated with shorter overall survival and poorer outcomes after ICI therapy. Singavi et al. reported that three patients in lung cancer and esophageal cancer harboring an 11q13 amplification experienced hyperprogression after treatment with ICIs (16). In our study, 11q13 amplification significantly affected ICI treatment benefits, regardless of CPS level. Our results are similar to previous observations that have implicated the immune-negative role of 11q13 amplification as a predictive biomarker.

We enrolled 121 patients in this study, which represents one of the largest HNSCC cohorts studied thus far. However, our study was a retrospective analysis, which limited the power of our findings. We are thus enrolling more patients for further studies to consolidate our conclusions. A prospective study is also planned to further examine the associations of certain gene aberrations with clinical efficacy after treatment with ICIs.

In conclusion, we showed that 11q13 amplification and *EGFR* mutations negatively correlated with PD-1 inhibitor therapy in a retrospectively analyzed clinical cohort. These genetic aberrations may serve as potential predictive biomarkers to identify patients who are unsuitable for immunotherapies, which will aid physicians in clinical decision-making. Therefore, further studies are required to confirm the utility of these biomarkers as predictors of treatment outcome in HNSCC.

## Data Availability Statement

The datasets presented in this study can be found in online repositories. The names of the repository/repositories and accession number(s) can be found in the article/**Supplementary Material**.

## Ethics Statement

The studies involving human participants were reviewed and approved by the ethics committee of Shanghai Ninth People’s Hospital (No. SH9H-2020-T257-1). Written informed consent for participation was not required for this study in accordance with the national legislation and the institutional requirements.

## Author Contributions

GZ, RL, SD, and LZ conceived and supervised the study. SD, LZ, CW, and YY drafted the manuscript. WJ, LY, and JL collected samples and clinical information. SW, DS, and XG analyzed the data. All authors have revised the manuscript and provided helpful advice. All authors read and approved the final manuscript.

## Funding

This study was supported by the Shanghai Anti-Cancer Research Foundation (H8001-004) and Clinical Research Plan of SHDC (No. SHDC2020CR4012) and WU JIEPING MEDICAL FOUNDATION (No. 320.6750.2021-01-34). The study sponsors had no role in the study design, data collection, analysis and interpretation of data or preparation of the manuscript.

## Conflict of Interest

Authors DS and XG are employed by Genecast Biotechnology.

The remaining authors declare that the research was conducted in the absence of any commercial or financial relationships that could be construed as a potential conflict of interest.

## Publisher’s Note

All claims expressed in this article are solely those of the authors and do not necessarily represent those of their affiliated organizations, or those of the publisher, the editors and the reviewers. Any product that may be evaluated in this article, or claim that may be made by its manufacturer, is not guaranteed or endorsed by the publisher.
