# Supplementary material for: EGFR Mutation and 11q13 Amplification Are Potential Predictive Biomarkers for Immunotherapy in Head and Neck Squamous Cell Carcinoma
Source: Front Immunol. 2022 Mar 16;13:813732. doi: 10.3389/fimmu.2022.813732 (PMC8965897; doi:10.3389/fimmu.2022.813732)
Supplement: Supplementary file 2 [file Table_2.docx]

Table S2. Analysis of patients treated with non-PD-1 inhibitor

| Characteristic | Clinical benefit (n, row%) | | p value (Fisher exact test) | Odds ratio (95% CI) |
| --- | --- | --- | --- | --- |
|  | CB | NCB |  |  |
| CPS |  |  |  |  |
| ≥ 1 | 19 (95.0) | 1 (5.0) | 0.018 | 13.57 (1.34, 137.46) |
| < 1 | 7 (58.3) | 5 (41.7) |  |  |
| 11q13 amplification |  |  |  |  |
| No | 14 (82.4) | 3 (17.6) | 1.000 | 1.17 (0.20, 6.89) |
| Yes | 12 (80.0) | 3 (20.0) |  |  |
| *EGFR* amplification |  |  |  |  |
| No | 17 (77.3) | 5 (22.7) | 0.637 | 0.38 (0.04, 3.75) |
| Yes | 9 (90.0) | 1 (10.0) |  |  |
| *EGFR* mutation |  |  |  |  |
| No | 26 (81.2) | 6 (18.8) | Not applicable | Not applicable |
| Yes | 0 (0) | 0 (0) |  |  |

CB: clinical benefit; NCB: non-clinical benefit; CPS: combined positive score;

11q13: CCND1_FGF3_FGF4_FGF19 or any one of them.
