# Supplementary figures and images for: EGFR Mutation and 11q13 Amplification Are Potential Predictive Biomarkers for Immunotherapy in Head and Neck Squamous Cell Carcinoma
Source: Front Immunol. 2022 Mar 16;13:813732. doi: 10.3389/fimmu.2022.813732 (PMC8965897; doi:10.3389/fimmu.2022.813732)

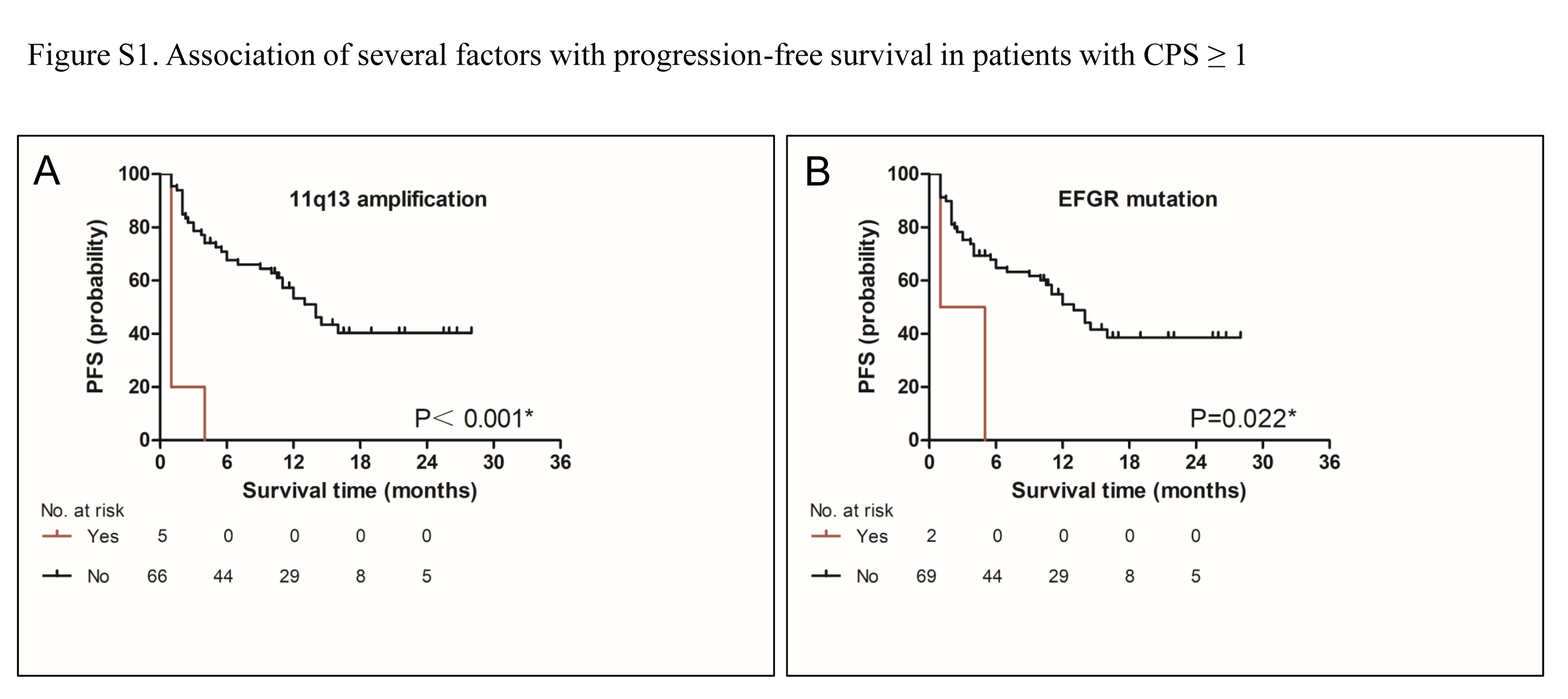

Supplement: Supplementary file 3 [file Image_1.tiff]

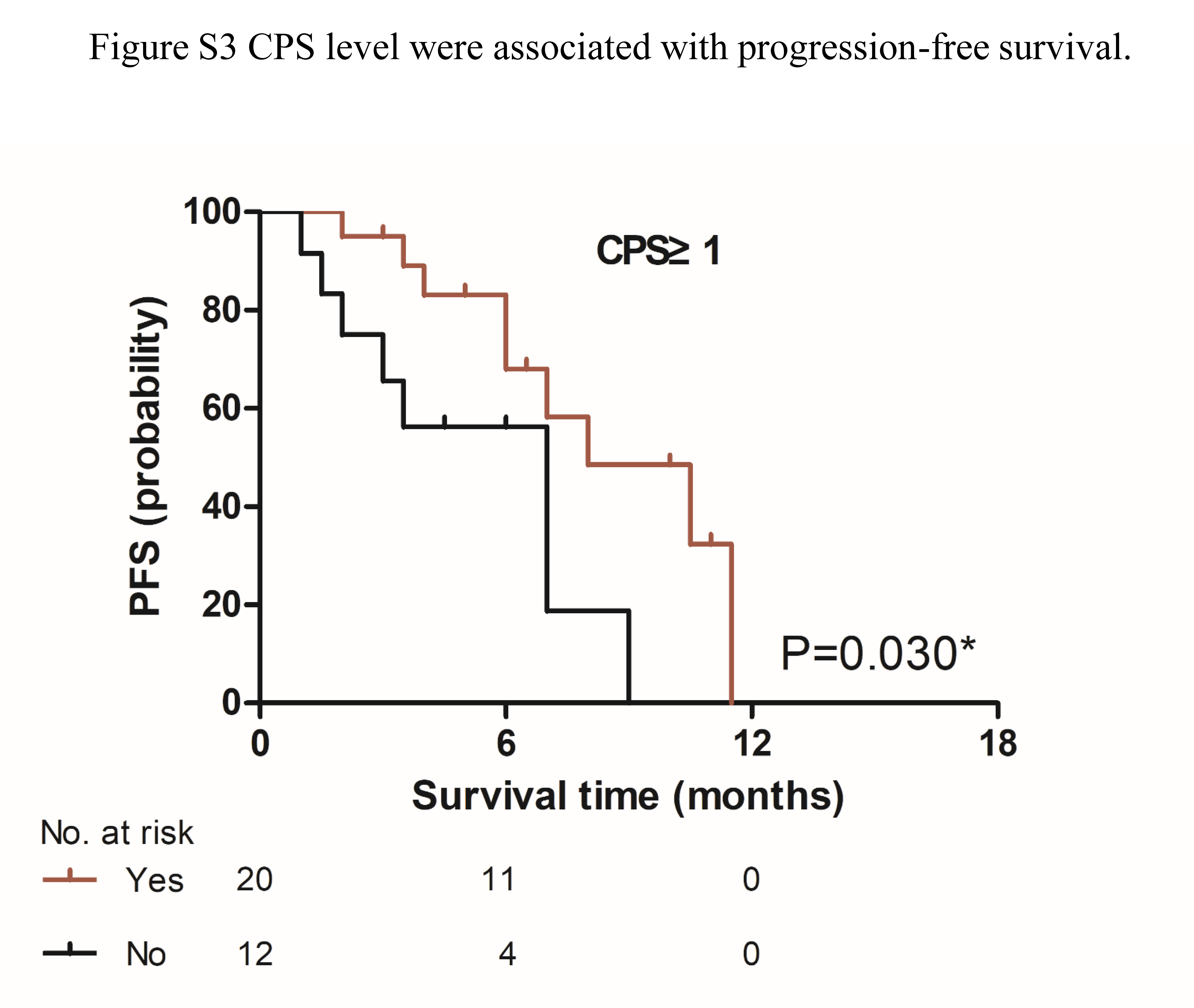

Supplement: Supplementary file 5 [file Image_3.tiff]
